# Asymptomatic wide complex tachycardia: a case report

**DOI:** 10.1186/1757-1626-2-47

**Published:** 2009-01-13

**Authors:** Siddharth Mukerji, Feras Aloka, Atul Khasnis

**Affiliations:** 1 Department of Internal Medicine, Michigan State University, East Lansing, Michigan, USA

## Abstract

Wide complex tachycardias are a commonly encountered entity in coronary care units, intensive care units and emergency departments. Though, these arrhythmias are potentially fatal, they need to recognized first and treated appropriately. Associated physical signs are helpful in this. We present a case of a 54-year-old-female who recently underwent placement of an implantable cardioverter-defibrillator for cardiomyopathy and developed tachycardia.

## Case presentation

**Figure 1:**
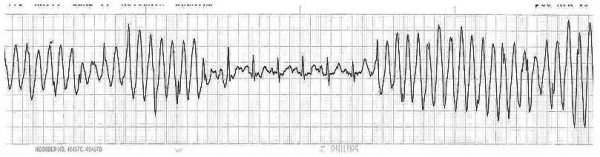
**Initial telemetry strip showing "wide QRS complexes" with intervening sinus rhythm**.

A 54-year-old female presented with shortness of breath (Class II-III NYHA) for one month. There was no associated chest pain, palpitations or dizziness. Her past history was significant for 3-vessel coronary artery bypass graft 10 years previously. Physical examination did not suggest any significant abnormalities. 2D-echocardiography revealed left ventricular ejection fraction of 28%. She underwent single chamber ICD placement for prevention of sudden cardiac death secondary to ischemic cardiomyopathy. The procedure was uneventful. The next day post-procedure, her telemetry strip showed this rhythm (Figure 1). Her vitals at that time were stable. What is the most likely diagnosis?

## Discussion

This is electrocardiographic (EKG) artifacts. (Table 1) EKG artifacts can be confused with ventricular tachycardia (VT) especially when of sufficient amplitude and duration. [1] This can lead to unnecessary investigations and at times, unindicated therapeutic interventions. Though the presence of a wide complex tachycardia (WCT) could alert one to the presence of VT, the absence of atrioventricular dissociation, fusion/capture beats, hemodynamic compromise (in view of the heart rate) make this possibility less likely.

**Table 1:** Selected differential diagnosis of wide complex tachycardia

Condition	Characteristics
Ventricular tachycardia (VT)	Presence of AV dissociation with more ventricular than atrial events, QRS duration more than 140 ms, fusion beats, capture beats
Supraventricular tachycardia with aberrancy	QRS duration of not more than 140 ms [7]
EKG artifacts	Hemodynamically stable, normal QRS complexes, "precipitating" cause viz. movements
Pre-excitation tachycardia	Presence of "delta wave", short PR interval
Ventricular fibrillation	No apparent rate, fibrillatory waves, absent pulse, unrecordable BP

**Figure 2:**
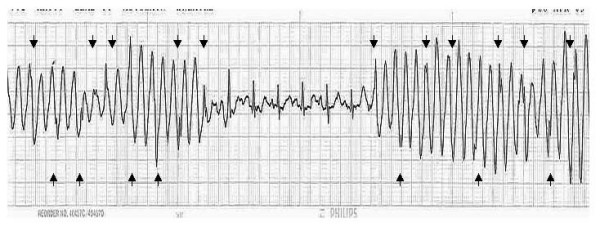
**True narrow QRS complexes amidst the "wide QRS rhythm"**.

Characteristics in favor of EKG artifacts include:

• Normal hemodynamic parameters during the event

• Presence of normal QRS complexes (arrows in Figure 2)

• Unstable baseline on the EKG

• Associated body movements [2]

Krasnow et al showed that the most likely causes of EKG artifacts that mimic VT are body movements and a poor skin-electrode contact. [3]

In our patient, the ICD implantation was done a day prior to this event. Routine device interrogation post-implantation did not reveal any abnormal events. In addition, the patient was noticed to have voluntary movements in the form of "shaking her leg" and examination revealed normal hemodynamic parameters. Serum electrolytes were within normal limits too. Hence, the presence of an asymptomatic patient with a normally functioning ICD not firing should alert the physician to the possibility of artifacts rather than an abnormal rhythm.

No treatment is indicated for this condition. Therefore, in modern day medicine it is imperative to differentiate artifacts from VT, and thus reduce health costs.

Ventricular tachycardia accounts for 80% of WCT. [4,5] In such patients, a history of structural heart disease, particularly of coronary artery disease or prior myocardial infarction, strongly suggests a diagnosis of VT.

SVT with aberrancy accounts for a relatively small number of patients with WCT. This tachycardia typically originates in atrial tissue and/or AV junction and utilizes the normal atrioventricular (AV) conduction system for ventricular activation. Aberrance occurs when there is delay or block in the His-Purkinje system during antegrade conduction of impulses over the normal AV fascicles. Essentially, all types of SVT with aberrant conduction can present as a WCT. Atrial tachycardias, atrioventricular nodal reentrant tachycardias, orthodromic reciprocating tachycardias are some of the common SVTs which can be associated with aberrance. Termination of WCT by adenosine, digoxin, calcium-channel blockers, beta-blockers or vagal maneuvers is suggests SVT. Grubb, however, showed that VT too could be terminated with carotid sinus stimulation. [6]

In ventricular fibrillation multiple foci take over from the ventricles and produce a disorganized, chaotic rhythm. The patient is considered pulse-less, with no blood pressure, requiring immediate intervention. VF is secondary to coronary artery disease, myocardial ischemia, myocardial infarction, cardiomyopathy, cardiac trauma, drug toxicity, hypoxia, and electrolyte imbalance.

Preexcited tachycardias are conducted antegradely over an accessory pathway (AP). Evidence for the presence of an AP can be manifest of the surface electrocardiogram (EKG) which can show intermittent or continuous presence of a delta wave associated with a short PR interval. The delta wave represents the part of the ventricular myocardium that is depolarized through the AP.

## Conclusion

Thus, it is imperative for a clinician to recognize an EKG artifact and differentiate it from other causes of WCT. This would prevent unwarranted interventions and minimize unnecessary procedures.

## Consent

Written informed consent was obtained from the patient for publication of this case report and accompanying images. A copy of the written consent is available for review by the Editor-in-Chief of this journal.

## Competing interests

The authors declare that they have no competing interests.

## Authors' contributions

All authors contributed equally in collecting patient data, chart review, and editing medical images. All authors read and approved the final manuscript.

## References

[B1] Knight BP Pelosi F Michaud GF Strickberger SA Morady F Clinical Consequences of Electrocardiographic Artifact Mimicking Ventricular Tachycardia N Engl J Med 1999 341 1270 1274 Oct 21, 1999 10.1056/NEJM199910213411704 10528037

[B2] Lin SL Wang SP Kong CW Chang MS Artifact simulating ventricular and atrial arrhythmia Jpn Heart J 1991 32 847 851 1811092 10.1536/ihj.32.847

[B3] Krasnow AZ Bloomfield DK Artifacts in portable electrocardiographic monitoring Am Heart J 1976 91 349 357 10.1016/S0002-8703(76)80220-7 1258734

[B4] Stewart RB Bardy GH Greene HL Wide complex tachycardia: misdiagnosed and outcome after emergency therapy Ann Intern Med 1986 104 766 771 3706928 10.7326/0003-4819-104-6-766

[B5] Steinman RT Herra C Scuger CD Wide complex tachycardia in the conscious adult: Ventricular tachycardia is the most common cause JAMA 1989 261 1013 1016 10.1001/jama.261.7.1013 2915409

[B6] Grubb BP Termination of ventricular tachycardia by carotid sinus stimulation Int J Cardiol 1989 23 397 399 10.1016/0167-5273(89)90201-5 2737783

[B7] Drew B Scheinman M ECG criteria to distinguish between aberrantly conducted supraventricular tachycardia and ventricular tachycardia Pacing Clinical Electrophysiol 1995 18 2194 2208 10.1111/j.1540-8159.1995.tb04647.x 8771133

